# Association between mental health and male fertility: depression, rather than anxiety, is linked to decreased semen quality

**DOI:** 10.3389/fendo.2024.1478848

**Published:** 2024-11-08

**Authors:** Yi Zhang, Bei Chen, Yaqin Wang, Cong Liu, Jiayi Sun, Zhimo Zhang, Liangzi Guan, Ke Xiao, Zhonghai Zhu, Jin Luo

**Affiliations:** ^1^ Reproductive Medicine Center, Renmin Hospital of Wuhan University, Wuhan, China; ^2^ Hubei Clinic Research Center for Assisted Reproductive Technology and Embryonic Development, Wuhan, China; ^3^ The Brain Cognition and Brain Disease Institute, Shenzhen Institute of Advanced Technology, Chinese Academy of Sciences, Shenzhen, Guangdong, China; ^4^ Faculty of Life and Health Sciences, Shenzhen Institute of Advanced Technology, Chinese Academy of Sciences, Shenzhen, Guangdong, China; ^5^ Shenzhen-Hong Kong Institute of Brain Science, Shenzhen Institute of Advanced Technology, Chinese Academy of Sciences, Shenzhen, Guangdong, China; ^6^ Department of Epidemiology and Biostatistics, School of Public Health, Xi’an Jiaotong University Health Science Center, Xi’an, Shaanxi, China

**Keywords:** depression, anxiety, sleep duration, semen quality, mental health

## Abstract

**Background:**

Infertility is increasingly recognized as a global health issue affecting couples of reproductive age, with male factors contributing to approximately 50% of infertility cases. However, the association between depression and anxiety-two of the most prevalent mental health conditions-and impaired male fertility remains a subject of ongoing debate.

**Methods:**

In this cross-sectional study, male participants seeking fertility counseling at an IVF clinic were recruited. Symptoms of depression and anxiety were assessed using the Patient Health Questionnaire-9 (PHQ-9) and the Generalized Anxiety Disorder-7 (GAD-7), respectively. Generalized linear regression models (GLMs) were employed to investigate the relationships between mental health status and semen parameters.

**Results:**

Status of depression was negatively associated with semen quality parameters, whereas no statistically significant association was recognized between anxiety and semen quality except that sperm concentration was decreased by 25.60 (95% CI, 1.226 to 49.965, *P*=0.040) ×10^6^/ml in moderate to severe anxiety group referring to normal group. Furthermore, when stratified by sleep duration, moderate to severe depression group showed a great decrease in progressive motility (PR), total motility, concentration and total sperm count (TSC) as referred to normal group in participants with sleep duration less than 7 hours.

**Conclusion:**

The present study revealed that depression rather than anxiety was a negative factor that affected semen quality, especially in individuals with shorter sleep duration.

## 1 Introduction

Infertility imposes a global burden on couples of reproductive age. In the United States, infertility affects approximately 15% of couples, while in China, the current prevalence is estimated to be 25% (1, 2). Male factors contribute solely to 20% of infertility cases but are responsible for about 50% of infertile couples (3). Insufficient sperm quality is the primary cause of male factor infertility. It’s noteworthy that human semen quality has been declining rapidly in last few decades (4, 5), accompanied by psychosocial consequences (6). In addition to the many known factors such as lifestyle and exposure to environmental toxins that can lead to a decline in sperm quality (7–9), there has been increasing attention in recent years on the impact of mental disorders on male fertility (10, 11).

Mental disorders, characterized by psychological, biological, or developmental abnormalities, are syndromes that lead to clinically significant changes in cognition, emotion control or behavior (12). Depression and anxiety are two of the most prevalent mental illness, with lifetime incidence of 17% and 12% respectively, leading to significant disease burden and disability (13, 14). These disorders could severely affect spermatogenesis, possibly through induction of suppression of gonadotropin inhibitory hormone that further inhibits hypothalamic-pituitary-testicular axis, leading to decreased testosterone biosynthesis (15). An inverse dose-response relationship has been reported between severity of depression and semen quality parameters including semen volume, total sperm count and motility (11, 16). However, an absence of compelling relationship was noted as well (17–20). While much of the existing literature focuses on depression’s effects on reproductive function, there remains a lack of large-scale investigation into the association between anxiety and semen quality (10). Furthermore, despite depression and anxiety are highly comorbid (21), few prior work has considered the independent impact of depression and anxiety on semen quality (22, 23).

Additionally, previous studies have suggested interactions between sleep indicators and mental health status, potentially due to shared neural regulatory pathways (24). For instance, literature has indicated a bidirectional relationship between sleep and depression, where patients experiencing depression often report sleep disturbances, which in turn may exacerbate the risk of developing various psychological disorders, including depression (25, 26). However, no prior studies have concurrently assessed the combined effects of depression, anxiety, and sleep quality on semen quality.

Given these research gaps, our study aims to clarify the influence of both depression and anxiety on semen quality among 1053 Chinese participants who provided semen samples while attending an IVF clinic for fertility counseling over a period of six months. We also seek to investigate the mediating effects of sleep quality on this relationship, hypothesizing that sleep may play a crucial role in understanding how mental health influences male fertility. By stratifying our analysis based on sleep habits, we hope to provide a comprehensive overview of how mental health and sleep interact to impact sperm quality, thereby enriching the current understanding of male infertility.

## 2 Materials and methods

### 2.1 Study design and participants recruitment

The observational study was carried out in Renmin Hospital of Wuhan University. This study is a cross-sectional analysis of male participants attending IVF clinic for fertility counseling, starting from January 2022 and ending in June 2022. Male individuals that meet the following criteria were included: (1) attending IVF clinic for fertility counseling; (2) aged between 20 and 45 years; (3) 2-7 days of abstinence; (4) no history of genetic diseases; (5) no presence of genitourinary tract diseases such as epididymitis, testicular injury, varicocele with or without previous treatment and inflammation in urinary genital tract; (6) no systemic diseases or severe organic diseases such as urogenital diseases. Participants were excluded if they had taken any psychotropic drugs in the past 3 months (27). The research was approved by the Ethical Committee of Renmin Hospital of Wuhan University (approval No. WDRY2022-K026). All participants signed informed consent before enrollment.

### 2.2 Semen quality tests

Semen samples were provided by masturbation and delivered to andrology laboratory immediately. After liquefied at 37°C, samples were analyzed for semen volume, progressive motility (PR), non-progressive motility (NP), total motility, sperm concentration and total sperm count (TSC) according to manual of World Health Organization “WHO Laboratory Handbook Examining and Processing Human Semen” (fifth edition) (28). Sperm morphology was assessed using Diff-Quik staining method and observed under ×100 magnification. Semen parameters were further dichotomized as normal or abnormal according to the reference limits in the WHO manual. Briefly, the cut-off values for semen volume, PR, total motility, concentration, TSC, and morphology were 1.5 mL, 32%, 40%, 40×10^6^/mL, 39×10^6^/ejaculate and 4%, respectively.

### 2.3 Mental status assessment

Self-reported questionnaires were utilized to assess symptoms of depression and anxiety. Depressive symptoms experienced over the last two weeks were evaluated by Patient Health Questionnaire 9 (PHQ-9), which contains 9 self-evaluative items and is a validated tool for identifying depression in individuals: (1) anhedonia, (2) depressed mood, (3) sleep disturbance, (4) fatigue, (5) appetite changes, (6) low self-esteem, (7) concentration problems, (8) psychomotor disturbances, and (9) suicidal ideation. A score from 0-3 represents the severity of each symptom. The total PHQ-9 scores can range from 0 to 27, with the following cut-off scores used to categorized the degree of depression: 0-4, no depression; 5-9, mild depression; 10-14, moderate depression; and 15 or above, severe depression. A provisional diagnosis of depression was made for individuals with a PHQ-9 score greater than 4, while a diagnosis of major depressive disorder (MDD) was indicated by a score of 10 or higher. Patients were subsequently divided into 3 groups according to PHQ-9 scores: normal (PHQ-9 score ≤ 4); mild depression (PHQ-9 score 5-9); moderate to severe depression (PHQ-9 score ≥ 10) (29). Symptoms of anxiety were evaluated using Generalized Anxiety Disorder Scale-7 (GAD-7), which includes 7 self-reported items. Each item is rated from 0-3 depending on the severity, resulting in a total score from 0-21. Cut-off values for the degree of anxiety were 5, 10 and 15, representing mild, moderate, and severe anxiety respectively. Provisional diagnosis of anxiety was considered with GAD-7 greater than 4 and generalized anxiety disorder (GAD) no less than 10. Participants were then grouped as normal (GAD-7 score ≤ 4), mild anxiety (GAD-7 score 5-9) and moderate to severe anxiety (GAD-7 score ≥10) (30). The Cronbach’s alpha coefficients of the PHQ-9 and GAD-7 was 0.839 and 0.891, respectively (31, 32).

### 2.4 Covariables

At enrollment, each participant completed a questionnaire in the presence of a research assistant after semen sample collection. Days of abstinence were recorded when semen samples were provided. For each individual, the following items were collected: age, height, weight, education levels (high school or below, college, postgraduate), smoking (former smoker or never, current regular or occasional) or alcohol-taking (yes, no), exercise (never, current occasional, current regular) and occupational exposure to health hazards (yes, no), frequency of exercise (never, current occasional, current regular) and marital status (unmarried, married, remarried). Body mass index (BMI) was computed as weight (kilograms) divided by height (meters) squared. In addition, participants were also asked to report their sleeping habits including sleep duration (≥7 hours, <7 hours), sleep chronotype represented by bedtime (before 12 pm, after 12 pm) and self-reported sleep quality (good, not good).

### 2.5 Statistical analysis

The study sample were described through continuous and categorical variables using mean (SD, standard deviation) and number (percentage), respectively. We used the complete case analysis for minimal issue of missing in our sample, which only had a variable (morphology) having missing numbers with a percentage of 1.13% (12/1053). A 2-sided p value less than 0.05 was considered as statistical significance. Statistical analyses were conducted using Stata 15.0 (Stata Corp, College Station, Texas, USA) and R software (version 4.4.1, https://www.r-project.org/).

In the analysis using Restricted Cubic Spline (RCS) to examine the relationship between PHQ-9 scores and key semen quality parameters, we found that there is a nonlinear correlation between the PHQ-9 scores and each variable (**Supplementary Figure S1**). Therefore, in subsequent analyses, we used the degree of depression/anxiety as a categorical variable and employed a GLM model to examine the associations between male mental health status (both depression and anxiety) and continuous semen quality parameters. To comprehensively assess the semen quality, we constructed a new outcome variable F1 using the factor score derived from principal component analysis for volume, progressive rate, total motility, concentration and morphology. The following covariables were adjusted including sociodemographic characteristics (age, BMI, education levels, marital status), lifestyle (smoking, alcohol-taking, exercise), occupational hazards exposure and sleeping habits (sleep duration, time to bed, sleep quality). Furthermore, we estimated the odds ratio and its 95% confidence interval (CI) for the association of mental status and aforementioned categorized semen quality parameters. In addition, the interaction term of mental health times sleep duration was included in the regression models to estimate the interaction P value and we repeated the analysis above stratified by sleep duration.

## 3 Results

### 3.1 Population characteristics

**Figure 1:**
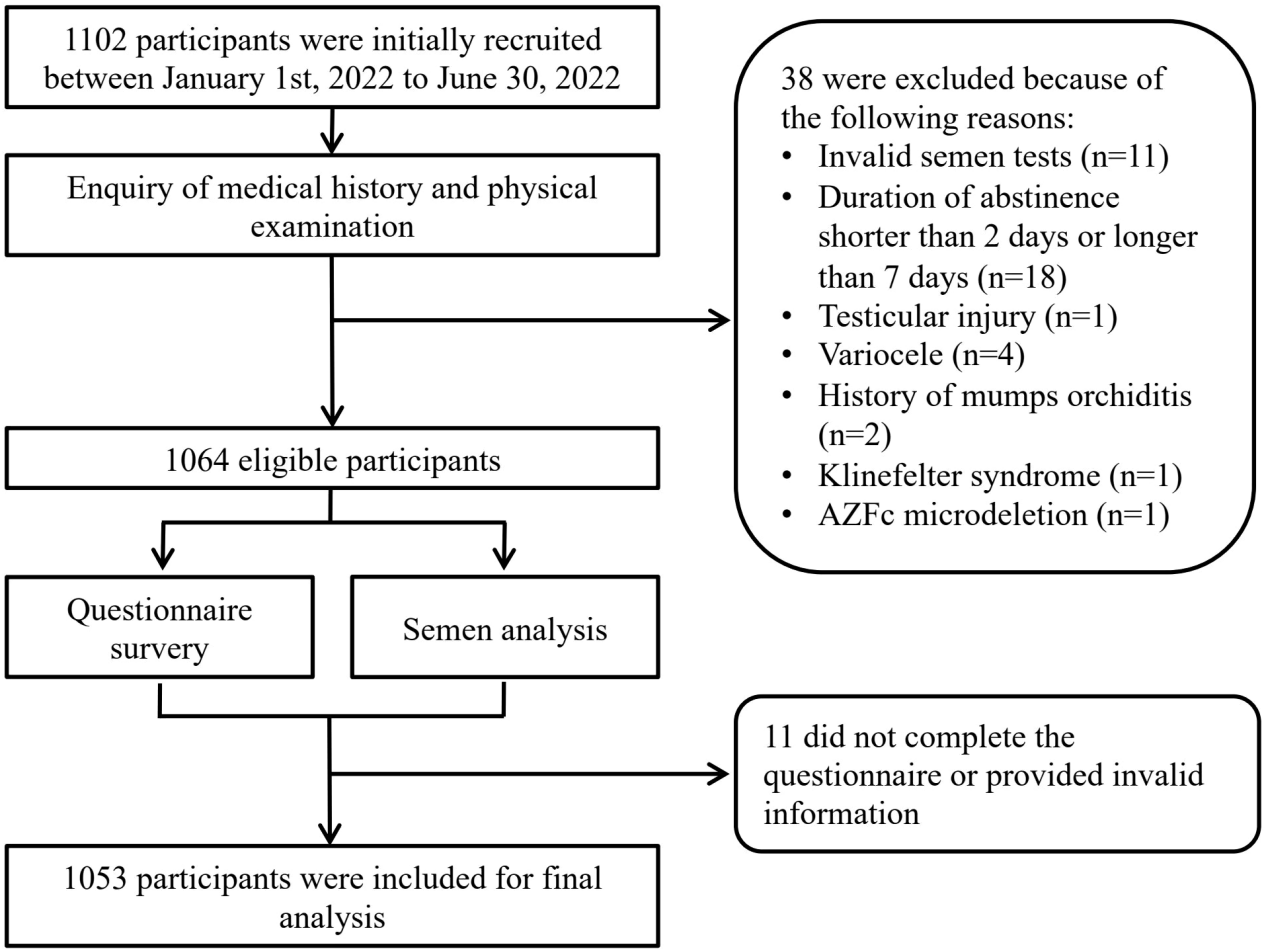
Flowchart of patient enrollment and selection.

A total of 1053 males were included for final analysis (**Figure 1**). The average age was 32.84 ± 4.37 years old and average BMI 24.32 ± 3.25 kg/m^2^. 359 (34.09%) participants has reported occupational exposure to more than one risk factors. The average PHQ-9 score was 2.64 ± 3.41 with 238 (22.6%) diagnosed with provisional depression, among which 198 (18.8%) were found to have symptoms of mild depression and 40 (3.8%) presenting symptoms of moderate to severe depression. Average GAD-7 score was 2.12 ± 2.97 and 174 (16.52%) were found to have symptoms of anxiety. Among participants provisionally diagnosed with anxiety, 29 (2.75%) were considered as moderate to severe anxiety. Other background characteristics and semen parameters were presented in **Table 1**.

**Table 1:** Descriptive characteristics of study population (n=1053).

Factors	No. (%)/Mean(SD)	Factors	No. (%)/Mean(SD)
Demographic characteristics			
Age(years)/Mean(SD)	32.84(4.37)	PHQ-9 scores	
Body mass index(kg/m^2^)/Mean(SD)	24.32(3.25)	Mean(SD)	2.64(3.41)
Education level		Anxiety	
High school or below	371(35.2)	Normal	879(83.48)
College	569(54.04)	Mild	145(13.77)
Postgraduate	113(10.73)	Moderate to severe	29(2.75)
Smoking		GAD-7 scores	
No	689(65.43)	Mean(SD)	2.12(2.97)
Yes	64(34.57)		
Drinking		**Semen parameters**	
No	714(67.81)	Days of abstinence	
Yes	339(32.19)	Mean(SD)	4.46 (1.51)
Exercise		Range	2-7
Barely not	183(17.38)	Volume(mL)	
Sometimes	715(67.90)	Mean(SD)	2.67(1.21)
Regular	155(14.72)	Range	0.3-9.8
Occupational exposure		Progressive motility (%)	
No	694(65.91)	Mean(SD)	41.87(21.60)
Yes	359(34.09)	Range	0-84.59
Bedtime		Non-progressive motility(%)	
Before 10 pm	56(5.3)	Mean(SD)	16.35(8.08)
10-12 pm	806(76.5)	Range	0.3-54.71
After 12 pm	131(18.1)	Total motility(%)	
Sleep duration		Mean(SD)	58.22(21.60)
≥7 hours	479(45.4)	Range	0.5-100.0
6-7 hours	480(45.5)	Concentration(×10^6^/mL)	
<6 hours	94(8.9)	Mean(SD)	82.34(64.95)
Sleep quality		Range	1.18-668.8
Good	847(80.4)	Total sperm count(×10^6^/ejaculate)	
Not good	206(19.5)	Mean(SD)	220.53(204.28)
Depression		Range	1.29-1556.4
Normal	815(77.40)	Morphology(%)	
Mild	198(18.80)	Mean(SD)	5.77(3.19)
Moderate to severe	40(3.80)	Range	0.18-19.26

^a^Data are missing for morphology (n=12). SD, standard deviation.

### 3.2 Association between male depression and semen quality

After adjusting for aforementioned covariables, GLM models showed a negative impact of depressive status on most of the semen quality parameters (**Table 2**). Compared with males without depression, the coefficient suggested that PR of participants with moderate to severe depression was 9.13 (95%CI, 3.273 to 14.995) % lower compared with those without depression (P=0.002). Similarly, total motility in moderate to severe depression group was 11.14 (95%CI, 4.046 to 18.240) ×10^6^/ml lower than those in normal group (P=0.002). Both individuals with mild and moderate to severe depression were prone to suffer from a decline in sperm concentration in comparison with normal participants, with an estimated decrease of 10.81(95%CI, 0.175 to 21.438) ×10^6^/ml and 47.71 (95%CI, 26.536 to 68.875) ×10^6^/ml respectively (P=0.046 and P<0.001). Total sperm count (TSC) was also decreased by 98.49 (95%CI, 32.112 to 164.865) ×10^6^/ejaculation in moderate to severe depressed participants with normal ones as reference (P=0.004). The synthetic variable F1 was also decreased by 0.25 (95%CI, 0.002 to 0.492) and 0.92(95%CI, 0.416 to 1.424) separately in mild depression and moderate to severe depression groups, referring to normal group (both P=0.048).

**Table 2:** Associations between depression status and semen quality in Chinese males.

GLM					Logistic regression		
	Normal	Mild depression	Moderate to severe depression		Normal	Mild depression	Moderate to severe depression
**N^a^**	815	198	40	**N^a^**	815	198	40
**Semen volume(mL)/Mean (SD)**	2.65(1.21)	2.70(1.21)	2.84(1.22)	**Semen volume<1.5 mL/N(%)**	77(9.45)	23(11.62)	3(7.50)
**Coefficient (95%CI)**	ref	0.18 (-0.02, 0.38)	0.43 (0.03, 0.82)*	**OR (95%CI)**	ref	0.95 (0.55, 1.65)	0.45 (0.12, 1.61)
**PR(%)/Mean (SD)**	42.73(17.57)	40.07(18.41)	33.25(19.01)	**PR<32%/N(%)**	240(29.45)	67(33.84)	22(55.00)
**Coefficient (95%CI)**	ref	-2.39 (-5.33, 0.56)	-9.13 (-15.00, -3.27)**	**OR (95%CI)**	ref	1.26 (0.87, 1.81)	3.33 (1.66, 6.66)**
**Total motility** **(%)/Mean(SD)**	59.28(21.20)	55.93(22.54)	47.93(21.93)	**Total motility<40%** **/N(%)**	171(20.98)	51(25.76)	16(40.00)
**Coefficient(95%CI)**	ref	-2.86 (-6.42, 0.71)	-11.14 (-18.24,-4.05)**	**OR (95%CI)**	ref	1.43 (0.96, 2.13)	3.20 (1.56, 6.58)**
**Concentration(×10^6^/mL)/Mean (SD)**	86.32(64.80)	74.67(67.49)	39.33(25.03)	**Concentration<40×10^6^/mL/N(%)**	44(5.40)	18(9.09)	5(12.50)
**Coefficient (95%CI)**	ref	-10.81 (-21.44, -0.18)*	-47.71 (-68.88, -26.54)***	**OR (95%CI)**	ref	1.99 (1.06, 3.73)*	2.91 (0.98, 8.63)
**TSC(×10^6^/ejaculate)/Mean(SD)**	228.58(202.67)	208.83(219.72)	114.50(104.45)	**TSC<39×10^6^/ejaculate/N(%)**	75(9.20)	23(11.62)	5(12.50)
**Coefficient(95%CI)**	ref	-8.46 (41.80, 24.87)	-98.49 (-164.87, -32.11)**	**OR (95%CI)**	ref	1.17 (0.68, 2.01)	1.20 (0.42, 3.40)
**Morphology(%)** **/Mean (SD)**	5.83(3.26)	5.62(2.94)	5.37(2.93)	**Morphology<4%** **/N(%)**	281(34.82)	68(34.52)	15(40.54)
**Coefficient(95%CI)**	ref	-0.34 (-0.87, 0.20)	-0.70 (-1.80, 0.39)	**OR (95%CI)**	ref	1.00 (0.70, 1.44)	1.42 (0.69, 2.91)
**F1/Mean (SD)**	0.84(1.45)	-0.18(1.53)	-0.85(1.49)	**F1<0.026 (50th percentile)/N(%)**	386(47.83)	107(54.31)	27(72.97)
**Coefficient (95%CI)**	ref	-0.25 (-0.49, -0.00)*	-0.92 (-1.42, -0.42)***	**OR (95%CI)**	ref	1.23 (0.88, 1,73)	2.81 (1.30, 6.08)**

GLM, generalized regression model; PR, progressive motility; TSC, total sperm count; CI, confidence interval; OR, odds ratio; SD, standard deviation.

a. Data are missing for morphology and F1: Normal (n=8), Mild depression (n=1), Moderate to severe depression (n=3).

*P<0.05, **P<0.005, ***P<0.001.

Findings was consistent in logistic regression model. Compared with men without depression, the OR for moderate to severe depression group in terms of lower PR (<32%) and total motility (<40%) was 3.33 (95%CI, 1.66 to 6.66, P=0.001) and 3.2 (95%CI, 1.56 to 6.58, P=0.002), respectively. For men with mild depression, the OR was 1.99 (95%CI, 1.06 to 3.73, P=0.032) for a low concentration (**Table 2**).

### 3.3 Status of anxiety was not related to semen quality

The same analysis was applied to analyze the association between anxiety and semen parameters. As revealed by GLM model, sperm concentration decreased by 25.60 (95% CI, 1.226 to 49.965, P=0.040) ×10^6^/ml in moderate to severe anxiety group using normal group as reference. No statistically significant association was recognized in terms of other semen quality parameters (**Table 3**). Similar non-significant associations were observed for categorized semen quality (**Table 3**).

**Table 3:** Impact of anxiety on semen quality based on GLM and regression analysis.

GLM					Logistic regression		
	Normal	Mild anxiety	Moderate to severe anxiety		Normal	Mild anxiety	Moderate to severe anxiety
**N^a^**	879	145	29	**N^a^**	879	145	29
**Semen volume(mL)/Mean (SD)**	2.66(1.21)	2.72(1.07)	2.50(1.65)	**Semen volume<1.5 mL/N(%)**	86(9.78)	11(7.59)	6(20.69)
**Coefficient (95%CI)**	ref	0.14 (-0.08, 0.36)	0.07 (-0.38, 0.52)	**OR (95%CI)**	ref	0.56 (0.28, 1.12)	1.25 (0.45, 3.47)
**PR(%)/Mean (SD)**	42.28(17.75)	39.76(17.68)	39.82(22.36)	**PR<32%/N(%)**	266(30.26)	51(35.17)	12(41.38)
**Coefficient (95%CI)**	ref	-1.66 (-4.93, 1.61)	-1.75 (-8.47, 4.98)	**OR (95%CI)**	ref	1.19 (0.80, 1.78)	1.73 (0.78, 3.82)
**Total motility** **(%)/Mean(SD)**	58.67(21.36)	56.17(22.39)	54.85(24.78)	**Total motility<40%/N(%)**	189(21.50)	39(26.90)	10(34.48)
**Coefficient(95%CI)**	ref	-1.25 (-5.22, 2.71)	-2.56 (-10.71, 5.59)	**OR (95%CI)**	ref	1.35 (0.87, 2.07)	2.28 (0.99, 5.23)
**Concentration(×10^6^/mL)/Mean (SD)**	84.65(66.56)	73.98(54.89)	54.22(52.21)	**Concentration<40×10^6^/mL/N(%)**	54(6.14)	8(5.52)	67(6.36)
**Coefficient (95%CI)**	ref	-8.29 (-20.14, 3.56)	-25.60 (-49.97, -1.23)*	**OR (95%CI)**	ref	0.90 (0.40, 2.02)	2.96 (0.99, 8.89)
**TSC(×10^6^/ejaculate)/Mean(SD)**	225.67(205.38)	203.81(193.50)	148.60(212.29)	**TSC<39×10^6^/ejaculate/N(%)**	81(9.22)	16(11.03)	6(20.69)
**Coefficient(95%CI)**	ref	-10.42 (-47.42, 26.58)	-46.94 (-123.04, 29.16)	**OR (95%CI)**	ref	1.16 (0.63, 2.13)	2.12 (0.78, 5.76)
**Morphology(%)** **/Mean (SD)**	5.77(3.21)	5.95(3.21)	5.02(2.10)	**Morphology<4%** **/N(%)**	307(35.21)	47(33.10)	10(20.69)
**Coefficient(95%CI)**	ref	0.11 (-0.48, 0.71)	-9.24 (-2.18, 0.33)	**OR (95%CI)**	ref	0.93 (0.62, 1.39)	1.12 (0.49, 2.57)
**F1/Mean (SD)**	0.03(1.47)	0.17(1.49)	-0.26(1.77)	**F1<0.026 (50th percentile)/N(%)**	428(49.08)	77(54.23)	15(55.56)
**Coefficient (95%CI)**	ref	-0.11 (-0.39, 0.16)	-0.22 (-0.79, 0.36)	**OR (95%CI)**	ref	1.11 (0.76, 1.62)	1.12 (0.50, 2.50)

GLM, generalized regression model; PR, progressive motility; TSC, total sperm count; CI, confidence interval; OR, odds ratio; SD, standard deviation.

a. Data are missing for morphology and F1: Normal (n=7), Mild depression (n=3), Moderate to severe anxiety (n=2).

*P<0.05.

### 3.4 Modifications of relationship between male mental health and semen quality by sleep duration

**Figure 2:**
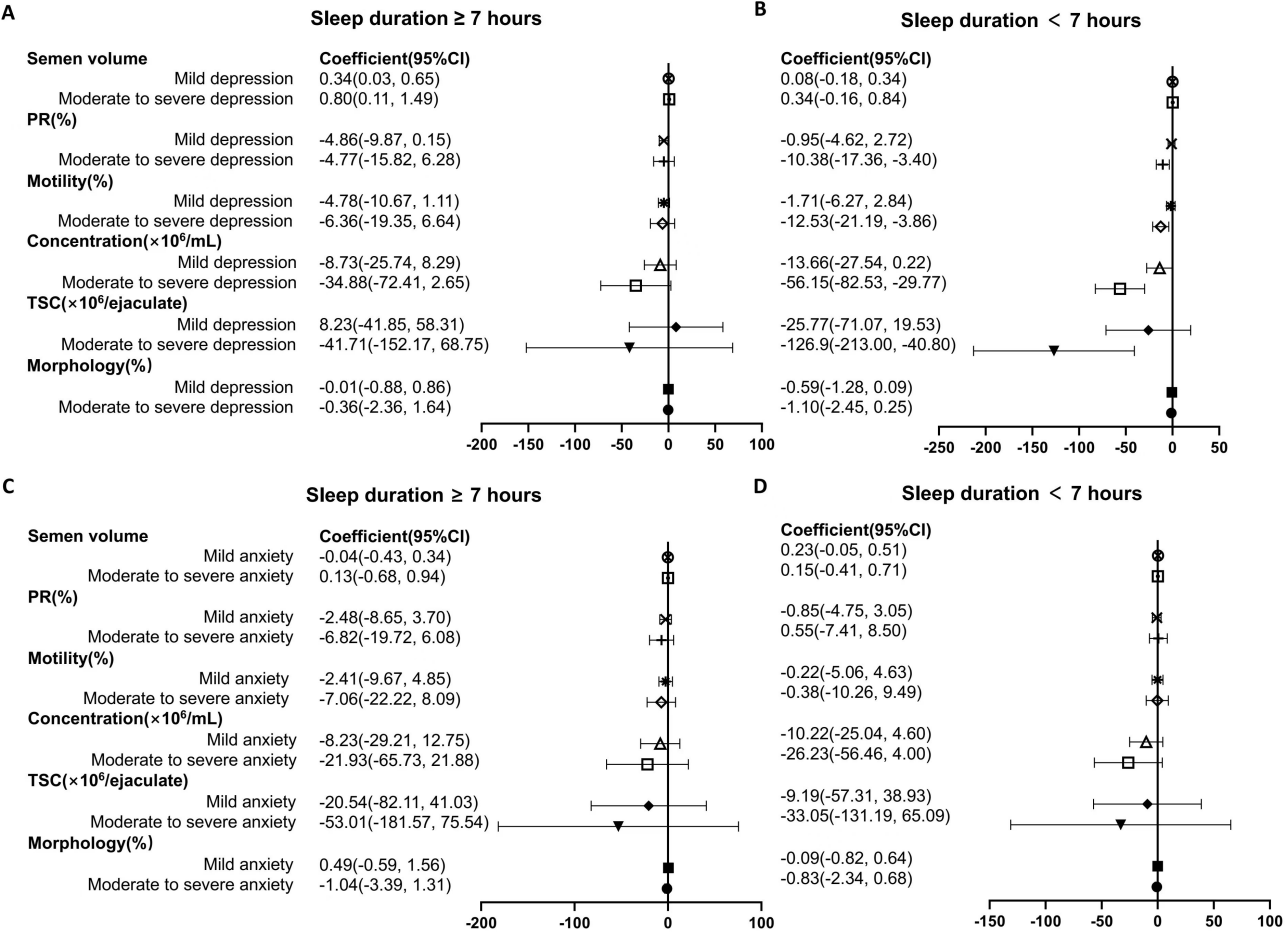
Forest plot summary of logistic regression analysis for the association between mental health status and semen parameters in individuals stratified by sleep duration. **(A, B)** The impact of depression on semen quality were analyzed in participants with sleep duration ≥ 7 hours **(A)** and < 7 hours **(B)**. **(C, D)** Association between anxiety status and semen parameters in individuals with sleep duration ≥ 7 hours **(C)** and < 7 hours **(D)**. PR, progressive rate; TSC, total sperm count. For each analysis, models are adjusted for age, body mass index, current smoking or alcohol-taking taking (yes, no), exercise (never, current occasional, current regular), occupational hazards exposure (yes, no), education level (high school or below, college, postgraduate), marital status (unmarried, married, remarried), time to bed (before 12 pm, after 12 pm), self-rated sleep quality (good, not good).

The impact of depression and anxiety on semen parameters were further analyzed stratified by sleep duration, and **Table 4** shows the corresponding interaction P value. In participants with sleep duration less than 7 hours, the status of moderate to severe depression was associated with a great decrease in PR (P_interaction_=0.510), total motility (P_interaction_=0.310), concentration (P_interaction_=0.869) and TSC (P_interaction_=0.271) as referred to normal group (**Figure 2**). In population with sleep duration ≥7 hours, no significant impact of depression on semen parameters was recognized. Nevertheless, the status of anxiety was not associated with decline in semen quality, stratified by sleep duration (**Figure 2**).

**Table 4:** Interaction *P* values between mental health status and sleep duration for semen quality in Chinese males.

Exposure	Volume	PR	Total motility	Concentration	TSC	Morphology
Depression	0.039	0.510	0.310	0.869	0.271	0.446
Anxiety	0.804	0.208	0.296	0.832	0.659	0.661

PR, progressive motility; TSC, total sperm count.

## 4 Discussion

In the present study, we found a negative impact of depression on semen quality parameters, particularly in terms of PR, total motility, sperm concentration and total sperm count (TSC). However, no statistically significant association was recognized between anxiety and semen parameters using the same models. Interestingly, when participants were stratified by sleep duration, depression was found to be negatively associated with sperm quality especially in patients with sleep duration less than 7 hours. In this group, moderate to severe depression was associated with declines in PR, total motility, sperm concentration as well as TSC.

Several studies have explored the correlation between depression and sperm quality, but findings are controversial. Ye et al. identified an inverse dose-response relationship between severity of depression and semen quality parameters, including semen volume, total sperm count and motility in healthy potential sperm donors (11). Another study found that lower semen volume was associated with a history of depression diagnosis and severe depressive symptoms (16). However, an absence of compelling relationship was noted as well (17–20). The observed inconsistencies in these studies may be attributed to variations in research populations or confounding variables. In our study, we found that the presence of depressive symptoms adversely effect on several semen quality parameters, including PR, total motility, sperm concentration, and total sperm count (TSC), as determined by generalized linear model (GLM) analysis. To comprehensively assess semen quality, a new outcome variable was derived from principal component analysis as F1, which was also decreased in both mild depression group and moderate to severe depression group. Consistent results were observed in the logistic regression model as well, indicating that depression was related to the decline in sperm quality, thereby contributing to male fertility issues.

Anxiety serves as a protective and adaptive response to various threats. However, excessive anxiety may have a negative impact on life quality and become a common chief complaint in psychiatry. Furthermore, excessive anxiety may be linked to neurological conditions or other systemic disorders (33). Recent studies have shown that anxiety is negatively associated with sperm parameters, including semen volume, count, concentration, and progressive motility, in infertile men and IVF patients (10, 34). Given the high comorbidity between depression and anxiety (21), it remains unclear whether depressive or anxious symptoms exert a greater influence on semen parameters. In the current study, symptoms of depression and anxiety were evaluated simultaneously using Patient Health Questionnaire 9 (PHQ-9) and Generalized Anxiety Disorder Scale-7 (GAD-7). Our results showed that, apart from a significant decrease in sperm concentration among participants with moderate to severe anxiety compared to those in the normal group, no statistically significant associations were recognized regrading other semen parameters in the GLM model. Similarly, no significant impact of anxiety was found on semen quality. These findings suggest that anxiety, compared with depression, may play a lesser role in affecting sperm quality.

Sleep disturbances were thought to affect fertility through possible endocrine pathways by altering several sex hormones, mainly testosterone in males (35, 36). Long-term sleep disorders can lead to a decline in immune function and may induce various physical diseases and psychological conditions, including anxiety and depressive symptoms (37). Several studies have investigated the association of sleep factors with human semen quality, but results were inconsistent. Chen et al. found that short sleep duration was associated with lower total and progressive sperm motility among 842 healthy men screened as potential sperm donors (38). In addition, research by Lauren et al. indicated that reduced sleep duration in men was associated with decreased fecundability (39). Nevertheless, Liu et al. (40) revealed that both short and long sleep duration were associated with decreased sperm count, survival rate, or motility among 981 healthy men. As far as we know, even though there was an interaction between sleep quality and mental status, no research has investigated the influence mental health on semen quality, taking into consideration of sleep duration. Interestingly, our findings revealed that depression was linked to sperm quality especially in patients with sleep duration less than 7 hours. In individuals experiencing moderate to severe depression, we observed that depression were associated with lower sperm quality, including PR, total motility, sperm concentration, volume, and total sperm count (TSC). In support of our findings, sleep quality has also been reported to correlate with sperm concentration, total count, and percentage of normal morphology among 953 healthy Danish men (41). Therefore, short sleep duration may partially mediate the relationship between depression and semen quality, providing valuable insights for fertility counseling in patients with depression.

### Strength and limitations

There might be some limitations in the present study. Due to the nature of observational study, we were only able to describe the association of different factors with semen parameters but were unable to define the causality. Additionally, like other cross-sectional studies, our findings are subject to unmeasured confounding factors and temporal relationships, which complicate causal inference. Our results may not be generalizable to the broader population, as all participants were recruited from an IVF clinic and the study was conducted at a single center. Furthermore, hormonal analysis and functional parameters such as DNA fragmentation index and seminal oxidative stress were not included in this study. Further research is needed to determine the relationship between male mental health and fertility. Moreover, sleep may represent a novel and innovative parameter to consider in the field of reproduction. More investigations are necessary to elucidate the relationship between sleep and fertility and how sleep may serve as a valuable modifiable target for infertility management.

## 5 Conclusion

Our study enhances the understanding of male infertility by demonstrating that depression, rather than anxiety, negatively impacts semen quality, particularly in individuals with shorter sleep duration. In the context of fertility counseling for depressed males, improving sleep quality, especially increasing sleep duration, may be an effective strategy to enhance semen quality.

## Data Availability

The original contributions presented in the study are included in the article/**Supplementary Material**. Further inquiries can be directed to the corresponding authors.

## Supplementary material

Supplementary Figure 1 Nonlinear regression analysis using restricted cubic spline to examine the relationship between PHQ-9 scores and four key semen quality parameters. **(A)** progressive motility (PR,%). **(B)** total motility (%). **(C)** sperm concentration (CON, millions/ml). **(D)** total sperm count (TSC, millions). Models are adjusted for age, body mass index, current smoking or alcohol-taking taking (yes, no), exercise (never, current occasional, current regular), occupational hazards exposure (yes, no), education level (high school or below, college, postgraduate), marital status (unmarried, married, remarried), time to bed (before 12 pm, after 12 pm), self-rated sleep quality (good, not good).

## References

[1] Thoma ME McLain AC Louis JF King RB Trumble AC Sundaram R. Prevalence of infertility in the United States as estimated by the current duration approach and a traditional constructed approach. Fertil Steril. (2013) 99:1324–31.e1. doi: 10.1016/j.fertnstert.2012.11.037 23290741 PMC3615032

[2] Zhou Z Zheng D Wu H Li R Xu S Kang Y. Epidemiology of infertility in China: a population-based study. BJOG. (2018) 125:432–41. doi: 10.1111/bjo.2018.125.issue-4 29030908

[3] Agarwal A Baskaran S Parekh N Cho CL Henkel R Vij S. Male infertility. Lancet. (2021) 397:319–33. doi: 10.1016/S0140-6736(20)32667-2 33308486

[4] Levine H Jorgensen N Martino-Andrade A Mendiola J Weksler-Derri D Jolles M. Temporal trends in sperm count: a systematic review and meta-regression analysis of samples collected globally in the 20th and 21st centuries. Hum Reprod Update. (2023) 29:157–76. doi: 10.1093/humupd/dmac035 36377604

[5] Lassen E Pacey A Skytte AB Montgomerie R. Recent decline in sperm motility among donor candidates at a sperm bank in Denmark. Hum Reprod. (2024) 39:1618–27. doi: 10.1093/humrep/deae115 PMC11291611 38834185

[6] de Vries CEJ Veerman-Verweij EM van den Hoogen A de Man-van Ginkel JM Ockhuijsen HDL. The psychosocial impact of male infertility on men undergoing ICSI treatment: a qualitative study. Reprod Health. (2024) 21:26. doi: 10.1186/s12978-024-01749-6 38374039 PMC10877778

[7] Yang W Duan Z Li G Geng H Gao Y Shen Q. Association of lifestyle and occupational exposure factors with human semen quality: a cross-sectional study of 1060 participants. Syst Biol Reprod Med. (2024) 70:150–63. doi: 10.1080/19396368.2024.2357348 38896558

[8] Fan S Zhang Z Wang H Luo L Xu B. Associations between tobacco inhalation and semen parameters in men with primary and secondary infertility: a cross-sectional study. Front Endocrinol (Lausanne). (2024) 15:1396793. doi: 10.3389/fendo.2024.1396793 38808116 PMC11130400

[9] Yang C Ning X Wang B Tian T Chen Y Ma L. Association between spectrum of mycotoxins and semen quality: A cross-sectional study in Beijing, China. J Hazard Mater. (2024) 476:135124. doi: 10.1016/j.jhazmat.2024.135124 38981237

[10] Pan Y Wang S Kang J Cao T Liu J Zhang L. Association between generalized anxiety symptoms and semen quality in infertile men: A multicentre study in North China. Andrologia. (2022) 54:e14449. doi: 10.1111/and.14449 35491407

[11] Ye YX Chen HG Sun B Chen YJ Duan P Meng TQ. Associations between depression, oxidative stress, and semen quality among 1,000 healthy men screened as potential sperm donors. Fertil Steril. (2022) 117:86–94. doi: 10.1016/j.fertnstert.2021.09.013 34656302

[12] Stein DJ Palk AC Kendler KS. What is a mental disorder? An exemplar-focused approach. Psychol Med. (2021) 51:894–901. doi: 10.1017/S0033291721001185 33843505 PMC8161428

[13] Bromet E Andrade LH Hwang I Sampson NA Alonso J de Girolamo G. Cross-national epidemiology of DSM-IV major depressive episode. BMC Med. (2011) 9:90. doi: 10.1186/1741-7015-9-90 21791035 PMC3163615

[14] Faravelli C Alessandra Scarpato M Castellini G Lo Sauro C. Gender differences in depression and anxiety: the role of age. Psychiatry Res. (2013) 210:1301–3. doi: 10.1016/j.psychres.2013.09.027 24135551

[15] Odetayo AF Akhigbe RE Bassey GE Hamed MA Olayaki LA. Impact of stress on male fertility: role of gonadotropin inhibitory hormone. Front Endocrinol (Lausanne). (2023) 14:1329564. doi: 10.3389/fendo.2023.1329564 38260147 PMC10801237

[16] Yland JJ Eisenberg ML Hatch EE Rothman KJ McKinnon CJ Nillni YI. A North American prospective study of depression, psychotropic medication use, and semen quality. Fertil Steril. (2021) 116:833–42. doi: 10.1016/j.fertnstert.2021.03.052 PMC8410630 33966888

[17] Coward RM Stetter C Kunselman A Trussell JC Lindgren MC Alvero RR. Fertility related quality of life, gonadal function and erectile dysfunction in male partners of couples with unexplained infertility. J Urol. (2019) 202:379–84. doi: 10.1097/JU.0000000000000205 PMC6686175 30835629

[18] Gurhan N Akyuz A Atici D Kisa S. Association of depression and anxiety with oocyte and sperm numbers and pregnancy outcomes during in *vitro* fertilization treatment. Psychol Rep. (2009) 104:796–806. doi: 10.2466/PR0.104.3.796-806 19708407

[19] Hjollund NH Bonde JP Henriksen TB Giwercman A Olsen J Danish First Pregnancy Planner Study T. Reproductive effects of male psychologic stress. Epidemiology. (2004) 15:21–7. doi: 10.1097/01.ede.0000100289.82156.8b 14712143

[20] Roopnarinesingh R Keane D Harrison R. Detecting mood disorders in men diagnosed with cancer who seek semen cryopreservation: a chance to improve service. Ir Med J. (2003) 96:104, 6–7. 12793470

[21] Bauer EA MacNamara A. Comorbid anxiety and depression: Opposing effects on the electrocortical processing of negative imagery in a focal fear sample. Depress Anxiety. (2021) 38:690–9. doi: 10.1002/da.23160 PMC8640943 33909324

[22] Pinarbasi FD Basar F Oguc AF. Effect of anxiety and depression levels on pregnancy outcome. Rev Assoc Med Bras (1992). (2024) 70:e20230922. doi: 10.1590/1806-9282.20230922 38451578 PMC10913781

[23] Walker Z Ernandez J Lanes A Srouji SS Ginsburg E Kathrins M. The effects of male anxiety and depression on IVF outcomes. Hum Reprod. (2023) 38:2119–27. doi: 10.1093/humrep/dead179 37690112

[24] Foster RG Peirson SN Wulff K Winnebeck E Vetter C Roenneberg T. Sleep and circadian rhythm disruption in social jetlag and mental illness. Prog Mol Biol Transl Sci. (2013) 119:325–46. doi: 10.1016/B978-0-12-396971-2.00011-7 23899602

[25] Qu Y Li T Xie Y Tao S Yang Y Zou L. Association of chronotype, social jetlag, sleep duration and depressive symptoms in Chinese college students. J Affect Disord. (2023) 320:735–41. doi: 10.1016/j.jad.2022.10.014 36270445

[26] Wang Z Zhu Y Li C Xin X Wang G Chen J. Correlation between physical exercise levels, depressive symptoms, and sleep quality in college students: Evidence from electroencephalography. J Affect Disord. (2024) 369:789–99. doi: 10.1016/j.jad.2024.10.043 39395679

[27] Beeder LA Samplaski MK. Effect of antidepressant medications on semen parameters and male fertility. Int J Urol. (2020) 27:39–46. doi: 10.1111/iju.14111 31542895

[28] World Health O. WHO laboratory manual for the examination and processing of human semen. 6th ed Vol. 2021. Geneva: World Health Organization (2021).

[29] Patel JS Oh Y Rand KL Wu W Cyders MA Kroenke K. Measurement invariance of the patient health questionnaire-9 (PHQ-9) depression screener in U.S. adults across sex, race/ethnicity, and education level: NHANES 2005-2016. Depress Anxiety. (2019) 36:813–23. doi: 10.1002/da.2019.36.issue-9 PMC6736700 31356710

[30] Spitzer RL Kroenke K Williams JB Lowe B. A brief measure for assessing generalized anxiety disorder: the GAD-7. Arch Intern Med. (2006) 166:1092–7. doi: 10.1001/archinte.166.10.1092 16717171

[31] Sun Y Kong Z Song Y Liu J Wang X. The validity and reliability of the PHQ-9 on screening of depression in neurology: a cross sectional study. BMC Psychiatry. (2022) 22:98. doi: 10.1186/s12888-021-03661-w 35139810 PMC8827244

[32] Gong L-L Xie X-L Liu S-T Hu W-H Niu Y-J Sun Y. Reliability and validity of generalized anxiety disorder 7-item scale in early pregnant women. Reprod Dev Med. (2022) 06:249–53. doi: 10.1097/RD9.0000000000000046

[33] Robinson OJ Pike AC Cornwell B Grillon C. The translational neural circuitry of anxiety. J Neurol Neurosurg Psychiatry. (2019) 90:1353–60. doi: 10.1136/jnnp-2019-321400 31256001

[34] Vellani E Colasante A Mamazza L Minasi MG Greco E Bevilacqua A. Association of state and trait anxiety to semen quality of in *vitro* fertilization patients: a controlled study. Fertil Steril. (2013) 99:1565–72. doi: 10.1016/j.fertnstert.2013.01.098 23414918

[35] Morssinkhof MWL van Wylick DW Priester-Vink S van der Werf YD den Heijer M van den Heuvel OA. Associations between sex hormones, sleep problems and depression: A systematic review. Neurosci Biobehav Rev. (2020) 118:669–80. doi: 10.1016/j.neubiorev.2020.08.006 32882313

[36] Gaml-Sorensen A Frolich MK Brix N Ernst A Bonde JPE Hougaard KS. Sleep duration and biomarkers of fecundity in young men: a cross-sectional study from a population-based cohort. Andrology. (2024) 12:1125–36. doi: 10.1111/andr.13560 37985426

[37] de Souza CM Hidalgo MP. Midpoint of sleep on school days is associated with depression among adolescents. Chronobiol Int. (2014) 31:199–205. doi: 10.3109/07420528.2013.838575 24156519

[38] Chen HG Sun B Chen YJ Chavarro JE Hu SH Xiong CL. Sleep duration and quality in relation to semen quality in healthy men screened as potential sperm donors. Environ Int. (2020) 135:105368. doi: 10.1016/j.envint.2019.105368 31830732

[39] Wise LA Rothman KJ Wesselink AK Mikkelsen EM Sorensen HT McKinnon CJ. Male sleep duration and fecundability in a North American preconception cohort study. Fertil Steril. (2018) 109:453–9. doi: 10.1016/j.fertnstert.2017.11.037 PMC5868973 29566862

[40] Liu MM Liu L Chen L Yin XJ Liu H Zhang YH. Sleep deprivation and late bedtime impair sperm health through increasing antisperm antibody production: A prospective study of 981 healthy men. Med Sci Monit. (2017) 23:1842–8. doi: 10.12659/MSM.900101 PMC5402839 28412762

[41] Jensen TK Andersson AM Skakkebaek NE Joensen UN Blomberg Jensen M Lassen TH. Association of sleep disturbances with reduced semen quality: a cross-sectional study among 953 healthy young Danish men. Am J Epidemiol. (2013) 177:1027–37. doi: 10.1093/aje/kws420 23568594

