# Supplementary figures and images for: Association between mental health and male fertility: depression, rather than anxiety, is linked to decreased semen quality
Source: Front Endocrinol (Lausanne). 2024 Nov 8;15:1478848. doi: 10.3389/fendo.2024.1478848 (PMC11581891; doi:10.3389/fendo.2024.1478848)

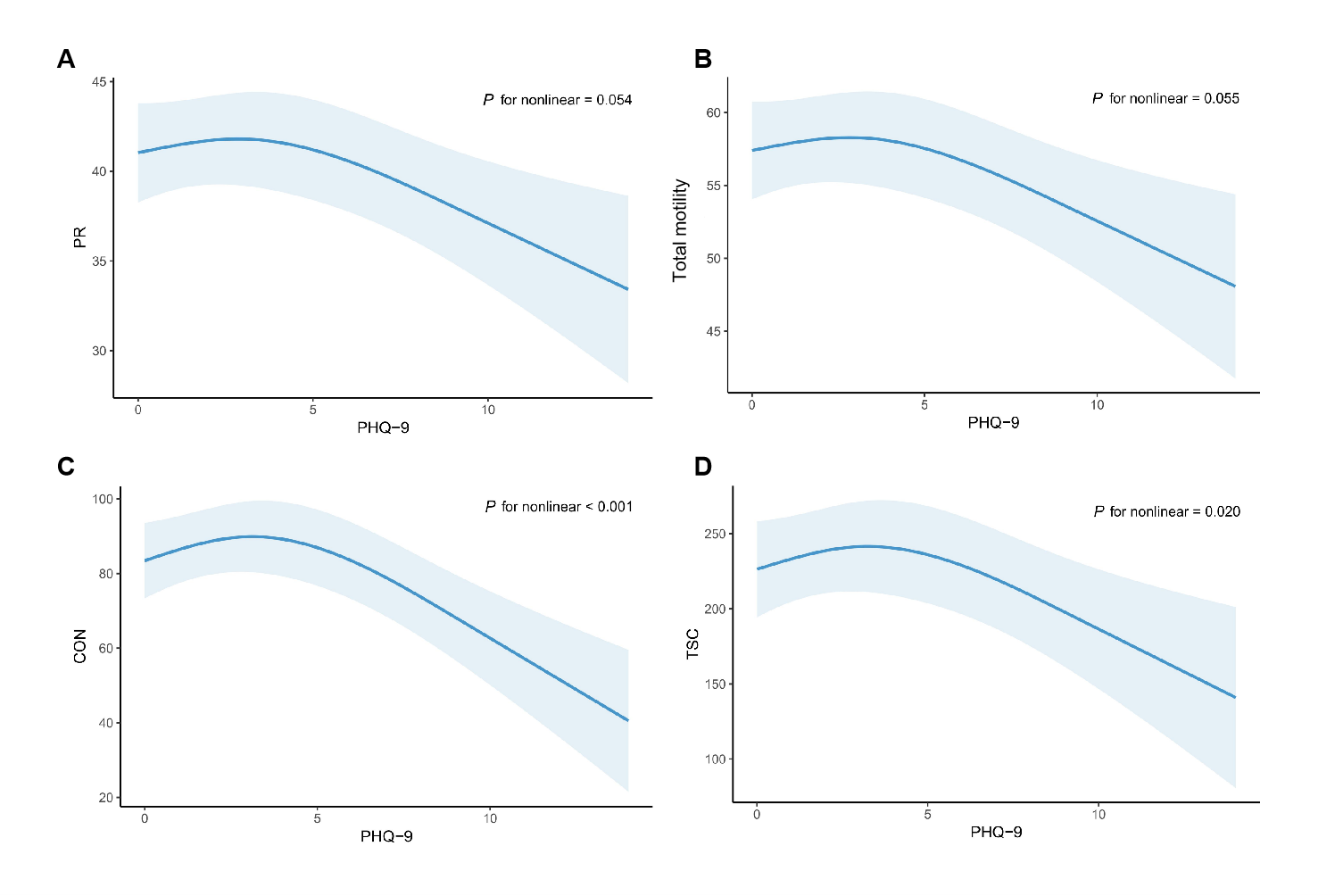

Supplement: Supplementary Figure 1 — Nonlinear regression analysis using restricted cubic spline to examine the relationship between PHQ-9 scores and four key semen quality parameters. (A) progressive motility (PR,%). (B) total motility (%). (C) sperm concentration (CON, millions/ml). (D) total sperm count (TSC, millions). Models are adjusted for age, body mass index, current smoking or alcohol-taking taking (yes, no), exercise (never, current occasional, current regular), occupational hazards exposure (yes, no), education level (high school or below, college, postgraduate), marital status (unmarried, married, remarried), time to bed (before 12 pm, after 12 pm), self-rated sleep quality (good, not good). [file Image1.tif]
